# Spatio-Temporal Comprehensive Measurements of Chinese Citizens’ Health Levels and Associated Influencing Factors

**DOI:** 10.3390/healthcare8030231

**Published:** 2020-07-25

**Authors:** Chenyu Lu, Shulei Jin, Xianglong Tang, Chengpeng Lu, Hengji Li, Jiaxing Pang

**Affiliations:** 1 College of Geography and Environmental Science, Northwest Normal University, Lanzhou 730070, China; jsl04050@163.com; 2 School of Architecture & Urban Planning, Lanzhou Jiaotong University, Lanzhou 730070, China; tangxl@mail.lzjtu.cn; 3 Institute for County Economy Developments & Rural Revitalization Strategy, Lanzhou University, Lanzhou 730000, China; lcp@lzu.edu.cn; 4 Information Center for Global Change Studies, Lanzhou Information Center of Chinese Academy of Sciences, Lanzhou 730000, China; lihengji@llas.ac.cn; 5 College of Earth and Environmental Sciences, Lanzhou University, Lanzhou 730000, China; pangjx@lzu.edu.cn

**Keywords:** health levels, comprehensive measurement, influencing factors, China, GIS

## Abstract

Health is the basis of a good life and a guarantee of a high quality of life. Furthermore, it is a symbol of social development and progress. How to further improve the health levels of citizens and reduce regional differences in citizens’ health status has become a research topic of great interest that is attracting attention globally. This study takes 31 provinces (municipalities and autonomous regions) of China as the research object. Through using GIS (Geographic Information System) technology, the entropy method, spatial autocorrelation, stepwise regression, and other quantitative analysis methods, measurement models and index systems are developed in order to perform an analysis of the spatio-temporal comprehensive measurements of Chinese citizens’ health levels. Furthermore, the associated influencing factors are analyzed. It has important theoretical and practical significance. The conclusions are as follows: (1) Between 2002 and 2018, the overall health levels of Chinese citizens have generally exhibited an upward trend. Moreover, for most provinces, the health levels of their citizens have improved dramatically, although some provinces, such as Tianjin and Henan, showed a fluctuating downward trend, suggesting that the health levels of citizens in these regions displayed a tendency to deteriorate. (2) The health levels of citizens from China’s various provinces showed clear spatial distribution characteristics of clustering, as well as an obvious spatial dependence and spatial heterogeneity. As time goes by, the degree of spatial clustering with regard to citizens’ health levels tends to weaken. The health levels of Chinese citizens have developed a certain temporal stability, the overall health status of Chinese citizens shows a spatial differentiation of a northeast–southwest distribution pattern. (3) The average years of education and urbanization rate have a significant positive effect on the improvement of citizens’ health levels. The increase of average years of education and urbanization rate can promote the per capita income, which certainly could help improve citizens’ health status. The Engel coefficient, urban–rural income ratio, and amount of wastewater discharge all pose a significant negative effect on the improvement of citizens’ health levels, these three factors have played important roles in hindering the improvements of citizen health.

## 1. Introduction

Health is the basis of a good life and a guarantee of a high quality of life. Furthermore, it is a symbol of social development and progress [1]. Due to the rapid development of economic growth and medical technology, the health levels of global citizens have greatly improved [2]. However, on account of the uneven distribution of power, income, products, and services, impoverished rural areas and vulnerable populations experience higher health risks, and obvious regional differences with regard to citizens’ health levels exist across or within countries [3,4]. The World Health Organization (WHO, Geneva, Switzerland) has stressed the need for reducing health differences between different regions or groups of people, and has declared that one of the main social goals of all governments is to enable all people to have an opportunity to enjoy good health [5]. In 2015, the United Nations Sustainable Development Summit formally adopted the “2030 Agenda for Sustainable Development”, and established Global Sustainable Development Goals (SDGs), for which almost every SDG is related to human health, and “health and well-being” is the basic premise and ultimate aim of every goal [6]. In 2017, the WHO elaborated on ten issues related to global health inequality. Thus, it can be seen that the health of citizens has become a global concern. Moreover, the question of how to further improve the health levels of citizens and reduce regional differences in citizens’ health status has become a research topic of great interest that is attracting attention globally [7,8,9,10].

Since its reform and opening-up, China has achieved rapid economic growth, with the accessibility of medical and health services significantly enhanced, and the health status of citizens greatly improved [11]. In recent years particularly, with substantial medical and health care reforms and the continuous increase of investment in medical and health resources, the health levels of its citizens have improved dramatically. However, significant differences with regard to citizens’ health levels still exist among different regions, and these are causing serious challenges to the goal of developing a harmonious society in China. Therefore, China is striving to continuously optimize and adjust. In 2016, the Chinese government formally passed the blueprint of “Healthy China 2030”, emphasizing the need to integrate health criteria into all policies, accelerate transformation in healthcare development, significantly improve health equity, and provide a solid and healthy foundation for achieving two centennial goals and the Chinese dream of the great rejuvenation of the Chinese nation [12]. In 2017, the President Xi Jinping delivered a report at the 19th National Congress of the Communist Party, stating that the implementation of a healthy China strategy and the emphasis on people’s health are important symbols of national prosperity. Therefore, it is necessary to improve national health policies and provide a complete range and full cycle of health services for all citizens [13]. Clearly, the health status of its citizens is very important to China. Accordingly, how to make a further improvement to its citizens’ health levels and reduce the health care disparities is also a research topic of great interest in China.

This study takes 31 provinces (municipalities and autonomous regions) of China as the research object (Hong Kong, Macau, and Taiwan are not included due to data insufficiency). Through using GIS (Geographic Information System) technology, the entropy method, spatial autocorrelation, stepwise regression, and other quantitative analysis methods, measurement models and index systems are developed in order to perform an analysis of the spatio-temporal comprehensive measurements of Chinese citizens’ health levels, with the associated influencing factors further analyzed. On the one hand, the present study can supplement and improve the current health geography research system, both theoretically and empirically, and help enrich the theoretical content of human geography and sustainable development with a certain theoretical significance. On the other hand, it can provide a scientific basis for China to further improve its citizens’ health levels and reduce regional differences, and offer theoretical support and a decision-making basis for the smooth implementation of Healthy China 2030. Therefore, the present study also has an important practical significance.

## 2. Literature Review

International research on the topic of citizen health began in the 1970s. After WHO proposed their global strategic goal of “health for everyone in 2000”, research on citizen health began all over the world. During this period, the research focus was on descriptions of differences in regard to citizens’ health status. For example, Grossman described the phenomenon that the overall health status would increasingly depreciate as the individual citizen ages [14]; Vladeck described the phenomenon that the health levels of poor people in American cities were generally lower than that of rich people [15]; and Grand [16], Townsend [17], Smith et al. [18], and other scholars believed that differences in citizens’ health levels represented a widespread phenomenon. In the 1980s and 1990s, the research focus turned to the interpretation of how factors, such as income, social status, the medical environment, health environment, and other social and economic factors, might lead to differences in citizens’ health levels. For example, Macintyre analyzed the British Health Report (August 1980) and discovered that mortality differences were socioeconomically related, and higher social classes generally displayed lower mortality rates [19]; Perrin et al. suggested that the key to improving citizen health was to enhance the quality of medical and health services [20]; and Ityavyar concluded that class inequality poses the most serious threat to the health of Nigerian citizens [21]. Since the 21st century, differences in citizens’ health levels across different regions and the analysis of associated influencing factors have gradually become the focus of a lot of research. For example, Murray et al. [22], Willems et al. [23], Skaftun et al. [24], and other scholars used health indices such as mortality and morbidity to analyze differences in citizens’ health levels across different regions of the world; and Brunello et al. [25], Gallardo et al. [26], Addison et al. [27], Brown et al. [28], and other scholars used health indices such as life expectancy, cancer mortality, obesity, and weight problems to measure differences in citizens’ health levels across different regions. They further analyzed the influence of education, economy, family environment, and other factors on citizens’ health levels.

Domestic research on citizens’ health levels began in the 1980s and followed the international research trend. Between the 1990s and the beginning of the 21st century, Chinese scholars focused on describing differences in citizens’ health levels and health services among different regions. For example, Yin et al. analyzed the health levels of poor rural citizens across provinces in China and revealed disparities in the aspects of provision, accessibility, and financing of health services [29]; Zhu et al. analyzed citizens’ health levels in rural areas of the Henan Province and found that differences existed within different regions with regard to citizen health and health service levels [30]; and Xie analyzed the health levels of urban and rural citizens across the country based on the data collected by China Health and Nutrition Survey and concluded that health disparities existed between the rich and poor in China [31]. Since the beginning of the 21st century, the relevant domestic research has gradually matured with some fruitful results. During this period, the relevant research content included the assessment of the health status of different populations across different regions and the analysis of associated influencing factors. For example, Li et al. [32], Yang et al. [33], Huang [34], and others scholars analyzed regional differences in citizens’ health levels and the associated influencing factors that caused such differences; Dai et al. [35], Wang et al. [36], Gong et al. [37], and others scholars selected different provinces as the research object and analyzed differences in citizens’ health levels and the associated influencing factors; and Liu et al. [38], Zhao et al. [39], Li et al. [40], and other scholars selected a particular population (e.g., the elderly or rural population) as the research object and analyzed differences in the citizens’ health levels of such populations and the associated influencing factors.

Overall, the relevant research has achieved some rich results, but there are some obvious shortcomings. Firstly, studies that apply GIS technology and spatial analysis models, and combine SDGs at the same time, in order to have a spatio-temporal comprehensive measurement (e.g., from temporal and spatial dimensions) of Chinese citizens’ health levels, associated evolution patterns and characteristics, are still very rare. Secondly, on the basis of mathematical models and from the perspective of the economy, society, and environment, a comprehensive analysis of influencing factors that affect citizens’ health levels is also lacking, and any relevant studies are still in the preliminary exploration stage. Therefore, the present study can reduce this knowledge gap.

## 3. Data and Methodology

### 3.1. Index System and Data Sources

SDGs aim to turn to the way of sustainable development, and pay attention to the sustainable development of environment, economy, and society. Among them, goal 3, “Ensure healthy lives and promote well-being for all at all ages”, is an important health-related goal, which is not only the premise foundation of other goals, but also the ultimate goal of other goals. So, based on the existing research results [41,42,43,44,45,46], the present study followed the principles of comprehensiveness, effectiveness, representativeness, and independence. With a combination of SDGs and China’s regional characteristics, our study selected relevant indices from three aspects, namely, citizens’ health status, the health environment, and health services and guarantees, and used them to develop a comprehensive evaluation index system that can fully reflect the health levels of Chinese citizens in a relatively comprehensive manner (Table 1). Through this comprehensive evaluation index system of Chinese citizens’ health levels based on SDGs, we can effectively measure the progress of health goals of SDGs in China, to provide reference for China to implement the 2030 Agenda for Sustainable Development and promote the comprehensive realization of SDGs in China.

According to the health production function proposed by Grossman, and based on the existing research results [38,39,47,48,49] with a combination of the situation in Chinese regions, the present study selected the appropriate indices from the economic, social, and environmental aspects to develop an index system of influencing factors that affect Chinese citizens’ health levels (Table 2).

The present study used 2002–2018 as the research period, with all data coming from the China Statistical Yearbook, China Health Statistics Yearbook, China Environmental Statistics Yearbook, Statistical Yearbook of provinces (municipalities and autonomous regions), Statistical Bulletin on the Development of Health and Family Planning, Statistical Bulletin on National Economic and Social Development, and other relevant statistical and literature materials (Tables S1 and S2).

### 3.2. Research Method

#### 3.2.1. Measurement of Citizens’ Health Levels

For the measurement of a comprehensive index system, the main methods to determine the index weight are subjective weighting method and objective weighting method. Subjective weighting method is a kind of method to determine the weight according to the subjective importance of the evaluator to each index, such as AHP (Analytic Hierarchy Process). The original information of the objective weighting method is derived from the objective environment, it determines the weight according to the information provided by each index, such as the entropy method [50,51]. So, in order to eliminate the influence of human subjective factors in weight determination, based on the information of each element in the comprehensive evaluation index system, the entropy method was applied to measure citizens’ health levels. The specific calculation steps were as follows [52]:

1. Data standardization:

(1) $$\mathrm{Positive}\;\mathrm{index}:\;z_{ij}=\frac{x_{ij}-x_{\min}}{x_{\max}-x_{\min}}$$

(2) $$\mathrm{Negative}\;\mathrm{index}:\;z_{ij}=\frac{x_{\max}-x_{ij}}{x_{\max}-x_{\min}}$$

2. Scale factor:

(3) $$Y_{ij}=\frac{x^{\prime }{}_{ij}}{\sum_{i=1}^{m}x^{\prime }{}_{ij}}$$

3. The calculation of the index information entropy:

(4) $$e_{j}=-k\sum_{i=1}^{m}\left(Y_{ij}\times \ln Y_{ij}\right),k=\frac{1}{\ln m},0\leq e_{j}\leq 1$$

Information entropy was originally proposed by Shannon. In the formula, 2 is used as the base, and the unit is bit. The base of logarithm in the formula is related to the unit of information entropy. For different research, different bases and units can be used and exchanged. In theoretical derivation, *e* is often used as the base, and the unit is Nat. So, based on the existing research results [53,54,55], in this study, *e* was used as the base. The constant *k* is related to the sample number m of the system. For a system with completely disordered information, the order degree is zero, and its entropy value is the largest. When the sample is in the state of complete disorder distribution, *k* = 1/ln (m). This is accordant with some other references [56,57].

1. The calculation of the information entropy redundancy:

(5) $$d_{j}=1-e_{j}$$

2. The weight of the index:

(6) $$W_{i}=d_{i}/\sum_{j=1}^{n}d_{j}$$

3. The calculation of the composite scores:

(7) $$H_{ij}=\sum_{j=1}^{n}z_{ij}W_{i}$$

In this equation, *z_ij_* represents the standardized values of *x_ij_*; *x_ij_* represents the actual values; *x_max_* and *x*_min_ refer to the maximum and minimum value of the same index, respectively, during different periods; m refers to the number of regions; *n* refers to the number of indices; and *H_ij_* refers to the health index.

#### 3.2.2. A Spatial Pattern Analysis of Citizens’ Health Levels

By using spatial autocorrelation models, we analyzed the spatial pattern of Chinese citizens’ health levels. Specifically, the global Moran’s I index was used to determine whether there is a statistical aggregation or dispersion in the distribution of citizens’ health levels based on the following formula [58]:

(8) $$I=\frac{\sum_{i=1}^{n}\sum_{j=1}^{n}w_{ij}(x_{i}-\bar{x})(x_{j}-\bar{x})}{S^{2}\sum_{i=1}^{n}\sum_{j=1}^{n}w_{ij}}$$

In this formula, $\bar{x}=\frac{1}{n}\sum_{i=1}^{n}x_{i}$, $S^{2}=\frac{1}{n}\sum_{i=1}^{n}{\left(x_{i}-\bar{x}\right)}^{2}$, *n* is the number of evaluation objects, *x_i_* and *x_j_* represent the attribute value of the evaluation objects *i* and *j*, respectively, and *w_ij_* represents a spatial weight matrix.

The local Getis–Ord G* index was used to reflect the spatial dependence and heterogeneity of citizens’ health levels, and to explore the characteristics and patterns of local spatial autocorrelation. The formula was as follows [59]:

(9) $$G_{i}^{*}(d)=\sum_{i=1}^{n}w_{ij}(d)x_{i}/\sum_{i=1}^{n}x_{i}$$

In this formula, the value of $G_{i}^{*}(d)$ is statistically positive, suggesting that the surrounding values of the region *i* are relatively high and it belongs to a hotspot category. Otherwise, it belongs to a coldspot category. *x_i_* represents the observed value of the region *i*, and *w_ij_* represents a spatial weight matrix.

#### 3.2.3. An Analysis of the Influencing Factors that Affect Citizens’ Health Levels

A multiple linear regression was used to develop the model, and then variables were selected following the stepwise regression approach. The formula was as follows [60]:

(10) $$Y=\beta_{0}+\beta_{1}X_{1}+\beta_{2}X_{2}+\cdots +\beta_{i}X_{i}$$

where, *Y* is the response variable, $X_{1},X_{2},\cdots X_{i}$ are explanatory variables, *β*_0_ is the constant term, and $\beta_{1},\beta_{2},\cdots \beta_{i}$ are regression coefficients.

## 4. Results and Discussion

### 4.1. A Comprehensive Evaluation of Citizens’ Health Levels

**Figure 1:**
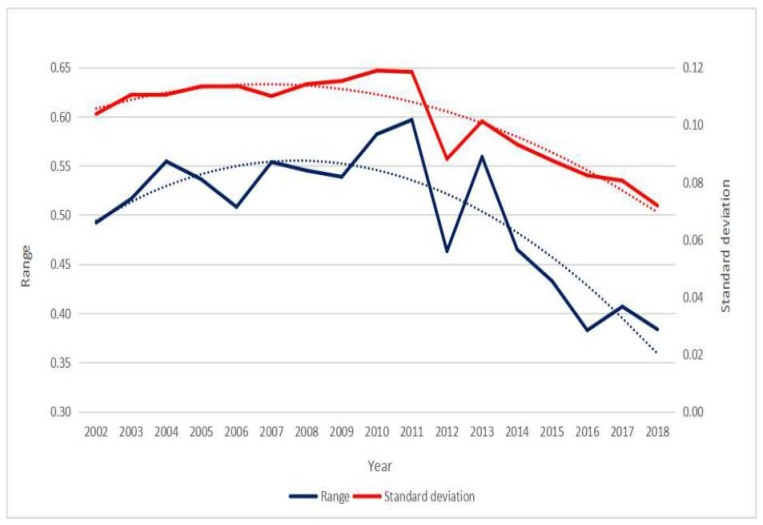
The change trend of the range and standard deviation of Chinese citizens’ health indices.

Between 2002 and 2018, the health indices of Chinese citizens generally remained in a relatively stable range (Table 3). Most provinces’ health indices showed a fluctuating rising trend, the citizens’ health levels in most provinces were gradually improved, and their health status has improved, but some provinces, such as Tianjin and Henan, displayed a fluctuating downward trend, suggesting that the health levels of citizens in these regions had a tendency to deteriorate, and this requires more attention. On the whole, the Chinese citizens’ health levels have been improved to a certain extent, which is developing in a benign direction. The range and standard deviation of health indices generally showed a trend of increasing first and then decreasing (Figure 1), suggesting that at the provincial level, differences in the health levels of Chinese citizens first increased and then decreased.

**Figure 2:**
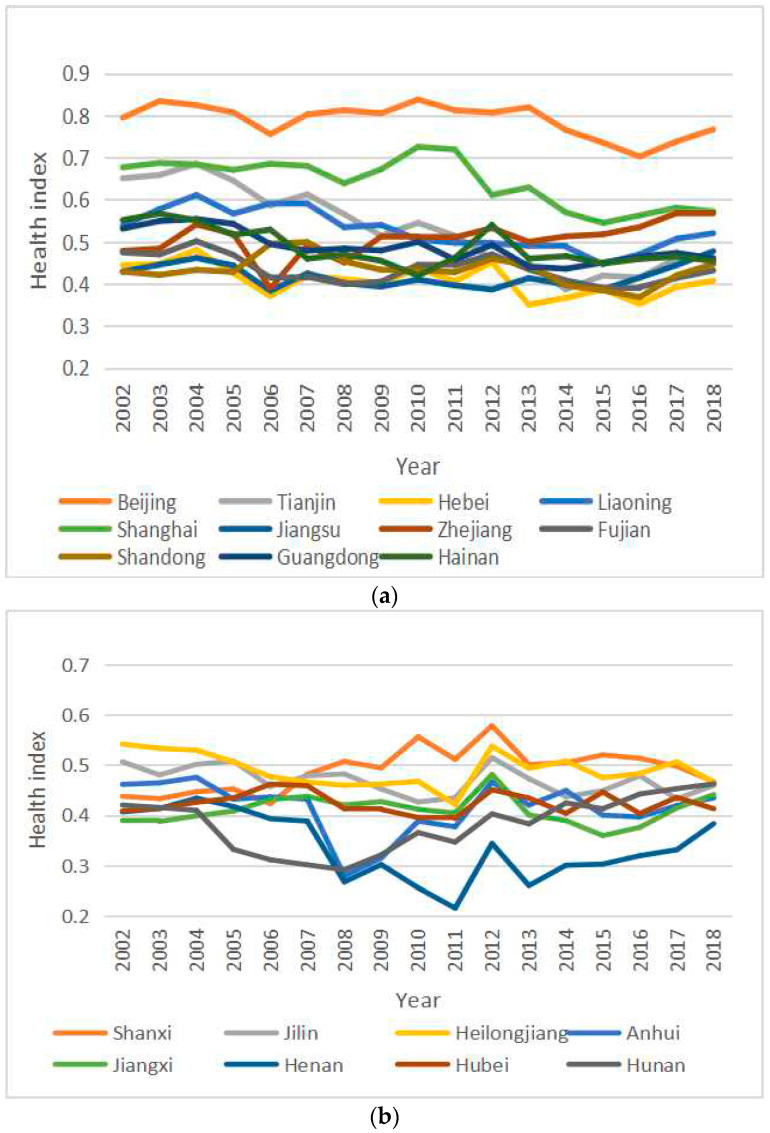

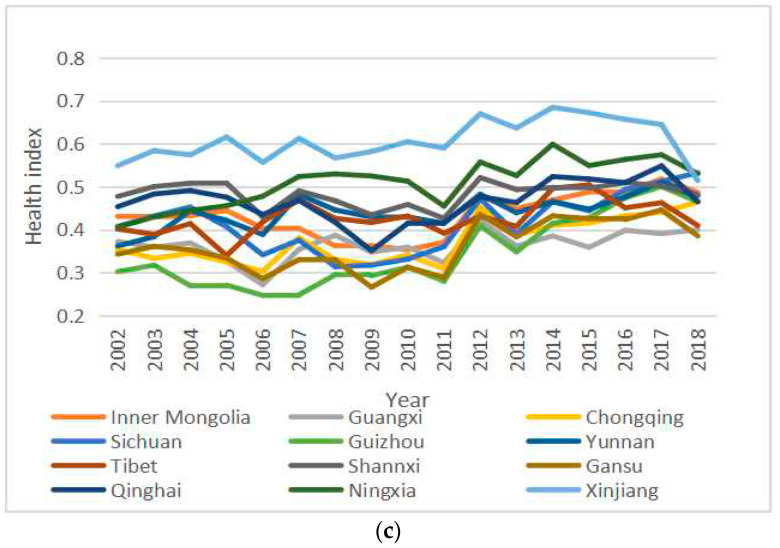
The change trend of Chinese citizens’ health indexes. (**a**) The change trend of citizens’ health indexes (the eastern region); (**b**) The change trend of citizens’ health indexes (the central region); (**c**) The change trend of citizens’ health indexes (the western region), (from the eastern, central, and western regions).

The present study followed the national method of categorizing the east, central, and west regions. Between 2002 and 2018, the overall health index of citizens in the eastern region was high, but the development trend is not optimistic. In particular, Beijing, Tianjin, Shanghai, and other big cities have repeatedly witnessed a continuous decline, partly due to high pressure of living and serious environmental pollution in such big cities (Figure 2a). The overall health index of citizens in the central region fluctuated greatly, but the development trend has gradually improved since 2013, which is related to the re-emphasis of the importance of implementing the Strategy for the Rise of Central Region of the state Council in 2012 (Figure 2b). The overall health index of citizens in the western region showed a fluctuating rising trend, and the health levels of citizens there have improved to a certain extent due to increasing subsidies and investment from the central government to the western region (Figure 2c).

**Figure 3:**
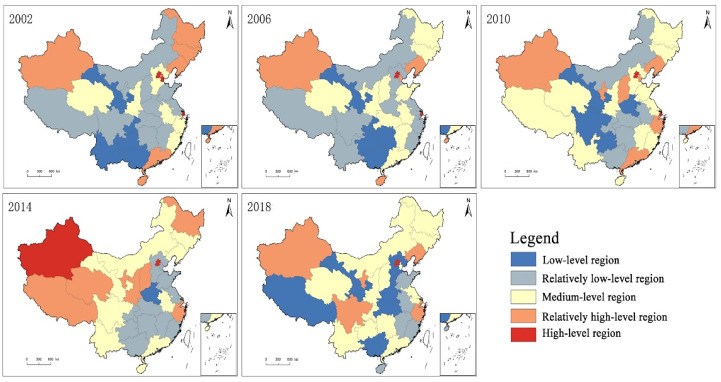
The spatial distribution of Chinese citizens’ health levels.

At the provincial level, in order to reflect the distribution characteristics of Chinese citizens’ health levels more intuitively, health indices that covered the years 2002, 2006, 2010, 2014, and 2018 were used. Based on ArcGIS software, the natural breakpoint method was applied so that 31 provinces (municipalities and autonomous regions) of China were categorized into five types: high-level region, relatively high-level region, medium-level region, relatively low-level region, and low-level region (Figure 3).

(1) In 2002, 5 provinces (including Gansu and Chongqing) belonged to the low-level category; this remained the same in 2006, but decreased to 4 in 2010, and only Henan remained at this low-level category in 2014. In 2018 however, 6 provinces (including Gansu and The Tibet Autonomous Region) belonged to the low-level category, and most of these were located in the central and western regions. (2) In 2002, 11 provinces belonged to the relatively low-level category, and most of these, for example, Sichuan and Jiangxi, were located in the central and western regions. In 2006, 2010, 2014, and 2018, the corresponding number of relatively low-level provinces was 9, 6, 11, and 5, respectively, and the distribution pattern shifted towards the eastern region. (3) In 2002, 6 provinces, including Shannxi and Anhui, belonged to the medium-level category. In 2006, 2010, and 2014, the corresponding number of medium-level provinces was 11, 12, and 9, respectively. In 2018, the number increased to 13, and most of these provinces were located in the central, western, and eastern coastal regions. (4) In 2002, 6 provinces, including Jilin and Guangdong, belonged to the relatively high-level category. In 2006, 2010, 2014, and 2018, the corresponding number of relatively high-level provinces was 4, 7, 8, and 6, respectively. The spatial distribution of these provinces was relatively scattered, with the eastern, central, and western regions all involved. (5) In 2002, 3 municipalities directly under the Central Government, such as Beijing, Tianjin, and Shanghai, belonged to the high-level category. In 2006, 2010, and 2014, the corresponding number of high-level provinces was 2, 2, and 2, respectively. In 2018, only Beijing remained in this high-level category.

From the perspective of the path of changes in citizens’ health levels, (1) between 2002 and 2006, 19.35% of the provinces moved to a higher category, whilst 25.81% moved to a lower category. The changing of the path between provinces was relatively straightforward (e.g., towards a higher or lower category). (2) Between 2006 and 2010, 38.71% of the provinces moved to a higher category, whilst 16.13% moved to a lower category. At this stage, more provinces moved to a higher category, and the first leapfrog-type shift phenomenon occurred. In particular, the Zhejiang Province moved from a relatively low-level category towards a relatively high-level category, whereas other provinces gradually moved to a higher or lower category. (3) Between 2010 and 2014, 32.26% of the provinces moved to a higher category, whilst 29.03% moved to a lower category. The changing of the path between provinces became more complicated, and the leapfrog-type shift phenomena were more obvious. Between 2014 and 2018, 22.58% of the provinces moved to a higher category, whilst 38.71% moved to a lower category. For the first time in the Tibet Autonomous Region, there was a phenomenon of a shift from a relatively high-level category towards a relatively low-level category, which can be explained by the medical infrastructure and ethnic habits of this region.

In recent years, other scholars have also conducted similar studies. For example, Yang et al. [61] analyzed temporal and spatial changes in China’s population health distribution and concluded that such differences, although they kept decreasing in recent years, were particularly significant at the provincial level. Zhao et al. [11] analyzed the evolution of regional differences in Chinese citizens’ health levels and discovered that their overall health levels have improved to some extent, especially in the western region. Hou [62] studied the degree of the regional health equity of Chinese citizens and reported that due to environmental pollution, life pressures, noise disturbance, and other unhealthy factors, the future health status and development trend of citizens in big cities were not expected to be good.

### 4.2. A Spatial Pattern Analysis of Citizens’ Health Levels

The global Moran’s I values of Chinese citizens’ health index in 2002, 2006, 2010, 2014, and 2018 were calculated (Table 4). All index values over these years were positive, with Z test values greater than the test threshold value of 2.58, and were significantly correlated at the level of 0.01, thereby passing the 99% confidence interval of statistical tests. It can be seen that health levels of Chinese citizens displayed a positive spatial autocorrelation and exhibited clustering features at the provincial scale. In other words, the health levels of Chinese citizens were spatially clustered (high/low regions) rather than randomly distributed. Provinces with relatively high levels of citizen health tended to be adjacent to provinces with similar high health levels, whereas provinces with relatively low levels of health tended to be distributed in proximity to provinces with similar low health levels. Judged from the changing trend of the global Moran’s I index, the overall health index showed a downward trend, suggesting that the degree of spatial autocorrelation has been somewhat reduced. In other words, the degree of spatial clustering distribution for provinces with either high or low levels of citizen health has been weakened to some extent.

**Figure 4:**
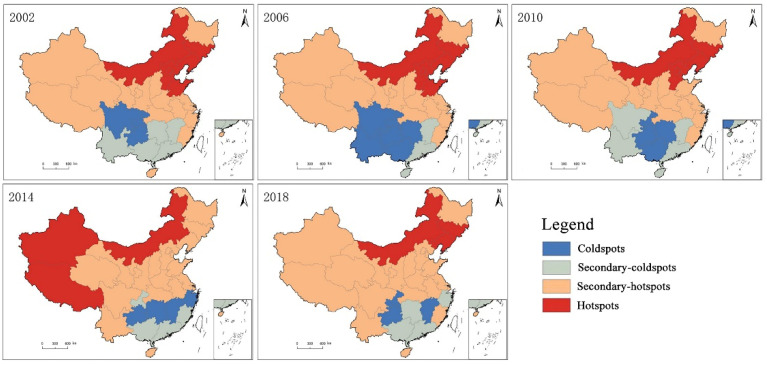
The spatial evolution of hotspots versus coldspots with regard to Chinese citizens’ health index.

This study was based on data collected in 2002, 2006, 2010, 2014, and 2018, and a local spatial autocorrelation analysis was performed with regard to Chinese citizens’ health levels. We used terms such as “coldspots”, “secondary-coldspots”, “secondary-hotspots”, and “hotspots” to measure the degree of correlation between the attribute values of each space unit and that of its adjacent space unit, and to reflect local spatial relationships (Figure 4).

(1) Between 2002 and 2006, the overall change was relatively small. Beijing, Jilin, and other hotspots remained unchanged and relatively stable. The Hainan Province was an exception, as it changed from a secondary-hotspot category to a secondary-coldspot category. Apart than that, the other secondary-hotspots remained unchanged. Meanwhile, the size of secondary-coldspots and coldspots kept contracting and expanding, respectively. Yunnan, Guangxi, and other provinces changed from secondary-coldspots to coldspots. (2) Between 2006 and 2010, the overall change was relatively small, and all hotspots remained unchanged except for the Shandong Province, which left the hotspots category. All secondary-hotspots also remained unchanged, expect for the addition of the Shandong Province, and these secondary-hotspots accounted for 51.61% of all the provinces. The secondary-coldspots and coldspots kept expanding and contracting, respectively, with Sichuan and Yunnan changing from the coldspot category to the secondary-coldspot category. (3) Between 2010 and 2014, the overall change was relatively large. The number of provinces in the hotspot category decreased from 6 to 3, and the geographic locations of such provinces shifted towards the central and western regions. Meanwhile, the proportion of provinces in the secondary-hotspot category further increased to 64.52%. Chongqing, Guangdong, and another four provinces were included in the secondary-coldspot category. Also, the number of provinces in the coldspot category remained unchanged, but the spatial distribution shifted from a block distribution pattern to a strip distribution pattern. (4) Between 2014 and 2018, the overall change was relatively large. The number of provinces in the hotspot category was unchanged. By contrast, Xinjiang and Tibet in the western region were not included in this category anymore, whereas Jilin and Liaoning in the central and eastern regions were included. As for the secondary-hotspot regions, the total number remained relatively stable, and these regions were also the largest, accounting for 67.74% of all provinces. Similarly, the number of provinces in the secondary-coldspot category also remained unchanged, although the number of provinces (including Chongqing) in the coldspot category was reduced to 3.

Overall, the health levels of Chinese citizens across various provinces exhibited some obvious spatial clustering characteristics, as well as a clear spatial dependence and spatial heterogeneity. Over time, regions of hotspots and coldspots generally followed the contraction trend, suggesting that the spatial clustering distribution of provinces with either high or low citizen health levels tended to weaken, which is in line with previous results based on the global Moran’s I index. The proportion of provinces in a stable status over all periods was higher than 50%, suggesting that Chinese citizens’ health levels have established certain temporal-stable distribution patterns. Specifically, Jilin, Liaoning, and some other provinces were in the stable hotspot category, whereas Chongqing, Guizhou, and some other provinces were in the stable coldspot category. The overall health status of Chinese citizens showed a spatial differentiation of a northeast–southwest distribution pattern. Furthermore, the number of provinces in the secondary hotspot category remained relatively stable, and this category was also the largest, as such provinces are widely distributed across China. By contrast, the number of provinces in the secondary coldspot category was relatively small, and such provinces, such as Guangdong and Guangxi, are mainly distributed in southern regions.

In recent years, other scholars have also conducted similar research, although the total number of such studies remains limited. For example, Wang [63] analyzed the temporal and spatial changes in China’s population health distribution and concluded that the spatial distribution of population health levels showed certain clustering features, rather than a random distribution pattern. Li et al. [32] analyzed regional differences of citizens’ health levels across various provinces in China and found out that spatial differences in health levels did exist, and certain temporal-stable distribution patterns were established.

### 4.3. An Analysis of the Influencing Factors that Affect Citizens’ Health Levels

In the present study, the health index was treated as the response variable, and the index of each influencing factor was considered as the explanatory variable. A regression analysis was performed, and appropriate variables were selected following the stepwise regression approach. The final model passed the F test and was statistically significant. It can be seen that the Engel coefficient X_2_, urban–rural income ratio X_3_, average years of education X_4_, urbanization rate X_5_, and wastewater discharge X_7_ each has a significant impact on the health levels of Chinese citizens (Table 5).

(11) $$Y=0.64419-0.00166X_{2}-0.01788X_{3}+0.02494X_{4}+0.00258X_{5}-0.03693X_{7}$$

The average years of education (X_4_) and the urbanization rate (X_5_) both had a positive effect on the improvement of citizens’ health levels and thus represent two important factors that could greatly promote this improvement. The increase of average years of education and urbanization rate can promote the per capita income, which certainly could help improve citizens’ health status. However, the positive effect of the average years of education was greater than that of the urbanization rate. The higher the average years of education, the more likely citizens would have more health-related knowledge. For example, citizens can have better living habits, make more healthy decisions in regard to disease prevention and treatment, and the health production function will be more efficient, all of which could promote the improvement of citizens’ health levels. The urbanization rate reflects the level of urban development. An increase in the level of urbanization will bring about fundamental changes in social organization, family relations, and lifestyles, which undoubtedly have many beneficial health effects and promote the development in a healthy direction. Additionally, it will help safeguard medical services and service guarantees, and ultimately promote the improvement of citizens’ health levels.

The Engel coefficient (X_2_), urban–rural income ratio (X_3_), and wastewater discharge (X_7_) all had negative impacts on the improvement of citizens’ health levels, of which wastewater discharge had the largest negative effect, followed by the urban–rural income ratio, with the Engel coefficient having the least negative effect. Regardless of their relative effects, these three factors have played important roles in hindering the improvements of citizen health. The negative impact of the deterioration of the environment on citizen health has become more prominent, out of which the water pollution problem is closely tied to citizen health. According to a WHO survey, 80% of human diseases are related to water pollution, which is also one of the major environmental challenges in China. Therefore, wastewater discharge has imposed a significant negative impact on the improvement of citizen health. An increase in the urban–rural income ratio would directly affect the balance of development between urban and rural areas, which in turn affects the equalization of public services among regions, resulting in weakening access to health products and services, and has a negative impact on the health of citizens. Therefore, the urban–rural income ratio has a significant negative effect on the improvement of citizen health. An increase in the Engel coefficient means that households’ expenditure on food consumption keeps increasing, which will inevitably crowd out other expenditures, resulting in insufficient investment in medical and health management, which would thus have an adverse effect on citizens’ health levels. Therefore, the Engel coefficient has a significant negative effect on the improvement of citizen health.

In recent years, other scholars have also conducted similar research, although such studies remain quite limited. For example, Xing [64] analyzed factors that affect citizens’ health levels and revealed that different variables such as economic and social development imposed different impacts on these levels. Nie et al. [65] analyzed the impact of social quality on citizens’ health status and concluded that to some extent, the four conditional factors of social quality all affected citizens’ health. Yao et al. [66] analyzed the health-related quality of life and associated influencing factors of Chinese citizens and found out that improving the education and income level of citizens, as well as their health behavior, was of great significance.

## 5. Conclusions

Between 2002 and 2018, the overall health levels of Chinese citizens have generally exhibited an upward trend. Moreover, for most provinces, the health levels of their citizens have improved dramatically, although some provinces, such as Tianjin and Henan, showed a fluctuating downward trend, suggesting that the health levels of citizens in these regions displayed a tendency to deteriorate. At the provincial level, certain differences in citizens’ health levels still exist, and such difference first increased and then decreased. The number of provinces with either high or low citizens’ health levels was relatively small, in comparison to the large number of provinces with medium or relatively low levels of citizen health, which were also widely distributed. By contrast, the spatial distribution of provinces with relatively high levels of citizen health was scattered. In recent years, citizens from Tianjin and Tibet have begun to display an unsteady development trend in regard to health levels, and there has been a leapfrog-type shift towards lower levels.

The health levels of citizens from China’s various provinces showed clear spatial distribution characteristics of clustering, as well as an obvious spatial dependence and spatial heterogeneity. Provinces with higher citizens’ health levels tended to be adjacent to other provinces with relatively high citizens’ health levels, and provinces with lower citizens’ health levels tended to be adjacent to other provinces with relatively low citizens’ health levels. As time goes by, the degree of spatial clustering with regard to citizens’ health levels tended to weaken. The health levels of Chinese citizens have developed a certain temporal stability. Specifically, Jilin, Liaoning, and other provinces are becoming stable hotspots, whereas places such as Chongqing and Guizhou are becoming stable coldspots. The overall health status of Chinese citizens shows a spatial differentiation of a northeast–southwest distribution pattern.

The Engel coefficient, urban–rural income ratio, average years of education, urbanization rate, and amount of wastewater discharge have posed a significant impact on Chinese citizens’ health levels. The average years of education and urbanization rate had a significant positive effect on the improvement of citizens’ health levels. The increase of average years of education and urbanization rate can promote the per capita income, which certainly could help improve citizens’ health status, with the positive effect of the average years of education greater than that of the urbanization rate. By contrast, the Engel coefficient, urban–rural income ratio, and amount of wastewater discharge all posed a significant negative effect on the improvement of citizens’ health levels. Specifically, wastewater discharge had the largest negative effect, followed by the urban–rural income ratio, with the Engel coefficient having the least negative effect. Regardless of their relative effects, these three factors have played important roles in hindering the improvements of citizen health.

## Figures and Tables

**Table 1:** The index system used for a comprehensive evaluation of Chinese citizens’ health levels.

Target Layer	Criteria Layer	Index Layer	SDGs Sources	Index Attributes
Health levels of Chinese citizens	Health status	Life expectancy per capita		+
		Population mortality	SDGs 3.4, 3.6	−
		Maternal mortality rate	SDGs 3.1	−
		Perinatal mortality	SDGs 3.2	−
		Statutory reporting of the incidence of infectious diseases in category A & B	SDGs 3.3	−
		Health literacy levels of citizens		+
	Health environment	Safe water popularizing rate	SDGs 6.1	+
		Rural sanitary toilet popularizing rate	SDGs 6.2	+
		The number of days when the air quality reaches or is better than type II	SDGs 11.6	+
		Landscaping ratio	SDGs 11.7	+
	Health services and guarantees	Hospitals per 10,000 people	SDGs 3.8	+
		Health technicians per 1000 people	SDGs 3.8	+
		Number of beds in medical and health institutions per 1000 people	SDGs 3.8	+
		Total health expenditure as a percentage of GDP	SDGs 3.8	+

SDGs: Sustainable Development Goals.

**Table 2:** The index system used for selecting influencing factors that affect Chinese citizens’ health levels.

Target Layer	Criteria Layer	Index Layer	A Brief Description of Each Index
Influencing factors that affect health levels of Chinese citizens	Economy	GDP per capita	X_1_
		Engel coefficient	X_2_
		Urban–rural income ratio	X_3_
	Society	Average years of education	X_4_
		Urbanization rate	X_5_
		Family size	X_6_
	Environment	Wastewater discharge	X_7_
		Solid waste discharge	X_8_
		Exhaust emission	X_9_

**Table 3:** The health indices of Chinese citizens.

Province	2002	2003	2004	2005	2006	2007	2008	2009	2010	2012	2013	2014	2015	2016	2017	2018
Beijing	0.79	0.83	0.82	0.81	0.76	0.8	0.81	0.8	0.84	0.81	0.82	0.77	0.74	0.7	0.74	0.77
Tianjin	0.65	0.66	0.68	0.64	0.59	0.61	0.57	0.51	0.54	0.46	0.45	0.39	0.42	0.41	0.46	0.46
Hebei	0.44	0.45	0.48	0.43	0.37	0.42	0.41	0.4	0.44	0.45	0.35	0.37	0.39	0.35	0.39	0.41
Shanxi	0.44	0.43	0.45	0.45	0.42	0.48	0.51	0.49	0.56	0.58	0.5	0.5	0.52	0.51	0.5	0.47
Inner Mongolia	0.43	0.43	0.43	0.44	0.4	0.4	0.36	0.36	0.35	0.48	0.45	0.47	0.49	0.49	0.52	0.49
Liaoning	0.54	0.58	0.61	0.57	0.59	0.59	0.53	0.54	0.51	0.5	0.49	0.49	0.45	0.47	0.51	0.52
Jilin	0.51	0.48	0.5	0.51	0.46	0.48	0.48	0.45	0.43	0.51	0.47	0.44	0.45	0.48	0.43	0.46
Heilongjiang	0.54	0.53	0.53	0.51	0.48	0.47	0.46	0.46	0.47	0.54	0.49	0.51	0.47	0.48	0.51	0.47
Shanghai	0.68	0.69	0.68	0.67	0.69	0.68	0.64	0.67	0.73	0.61	0.63	0.57	0.54	0.56	0.58	0.57
Jiangsu	0.43	0.45	0.46	0.44	0.38	0.42	0.4	0.39	0.41	0.39	0.41	0.4	0.39	0.41	0.44	0.48
Zhejiang	0.48	0.48	0.54	0.52	0.39	0.49	0.45	0.51	0.51	0.53	0.5	0.51	0.52	0.53	0.57	0.57
Anhui	0.46	0.46	0.48	0.43	0.44	0.43	0.28	0.31	0.39	0.47	0.42	0.45	0.4	0.4	0.42	0.43
Fujian	0.47	0.47	0.5	0.47	0.42	0.42	0.4	0.41	0.45	0.47	0.43	0.41	0.39	0.39	0.41	0.43
Jiangxi	0.39	0.39	0.4	0.41	0.43	0.44	0.42	0.43	0.41	0.48	0.4	0.39	0.36	0.38	0.41	0.44
Shandong	0.43	0.42	0.43	0.43	0.49	0.5	0.45	0.43	0.43	0.46	0.45	0.4	0.38	0.37	0.42	0.45
Henan	0.41	0.41	0.43	0.42	0.39	0.39	0.27	0.3	0.26	0.34	0.26	0.3	0.3	0.32	0.33	0.38
Hubei	0.41	0.41	0.43	0.43	0.46	0.46	0.41	0.41	0.4	0.45	0.43	0.4	0.45	0.4	0.44	0.41
Hunan	0.42	0.42	0.41	0.33	0.31	0.3	0.29	0.32	0.37	0.4	0.38	0.42	0.41	0.44	0.45	0.46
Guangdong	0.53	0.55	0.55	0.54	0.49	0.48	0.48	0.48	0.5	0.49	0.44	0.44	0.45	0.46	0.47	0.46
Guangxi	0.37	0.36	0.37	0.33	0.27	0.35	0.39	0.35	0.36	0.42	0.36	0.39	0.36	0.4	0.39	0.4
Hainan	0.55	0.57	0.55	0.52	0.53	0.46	0.47	0.45	0.42	0.54	0.46	0.47	0.45	0.46	0.46	0.45
Chongqing	0.35	0.33	0.35	0.33	0.3	0.38	0.33	0.32	0.34	0.45	0.39	0.41	0.42	0.43	0.44	0.46
Sichuan	0.41	0.43	0.45	0.41	0.34	0.38	0.31	0.32	0.33	0.47	0.39	0.47	0.44	0.49	0.51	0.53
Guizhou	0.3	0.32	0.27	0.27	0.25	0.25	0.3	0.29	0.31	0.41	0.35	0.42	0.43	0.47	0.5	0.47
Yunnan	0.36	0.38	0.44	0.42	0.39	0.48	0.45	0.43	0.43	0.48	0.44	0.47	0.45	0.48	0.51	0.48
Tibet	0.4	0.39	0.41	0.34	0.42	0.47	0.43	0.42	0.43	0.43	0.41	0.5	0.5	0.45	0.46	0.41
Shannxi	0.48	0.5	0.51	0.51	0.43	0.49	0.47	0.43	0.46	0.52	0.49	0.5	0.5	0.51	0.5	0.48
Gansu	0.34	0.36	0.35	0.33	0.29	0.33	0.33	0.27	0.31	0.44	0.38	0.43	0.43	0.42	0.45	0.39
Qinghai	0.45	0.48	0.49	0.48	0.44	0.47	0.42	0.35	0.41	0.48	0.46	0.52	0.52	0.51	0.55	0.47
Ningxia	0.41	0.43	0.44	0.46	0.48	0.52	0.53	0.52	0.51	0.56	0.53	0.6	0.55	0.56	0.57	0.53
Xinjiang	0.55	0.58	0.57	0.62	0.56	0.61	0.57	0.58	0.6	0.67	0.64	0.68	0.67	0.66	0.64	0.51

**Table 4:** The global Moran’s I values of Chinese citizens’ health index.

Year	2002	2006	2010	2014	2018
Moran’s I	0.487152	0.491435	0.479296	0.17159	0.295829
Z	7.257849	7.260378	7.263132	2.830804	5.466729

**Table 5:** The results of the stepwise regression.

Variable	Coefficient	Std. Error	T-Statistic	Prob.
C	0.64419	0.06120	10.53	0.000
X_2_	−0.00166	0.00065	−2.54	0.011
X_3_	−0.01788	0.00710	−2.52	0.012
X_4_	0.02494	0.00464	5.38	0.000
X_5_	0.00258	0.00035	7.36	0.000
X_7_	−0.03693	0.00249	−14.82	0.000
	R^2^	Adjusted R^2^	F-statistic	Prob(F-statistic)
	0.5912	0.5873	150.70	0.000

## Supplementary Materials

The following are available online at https://www.mdpi.com/2227-9032/8/3/231/s1, Table S1: The original data of measurement of citizens’health levels; Table S2: The original data of the influencing factors that affect citizens’health levels.
